# The *APOA1* p.Leu202Arg variant potentially causes autosomal recessive cardiac amyloidosis

**DOI:** 10.1038/s41439-024-00288-7

**Published:** 2024-08-16

**Authors:** Shusuke Yagi, Ryosuke Miyamoto, Masayoshi Tasaki, Hiroyuki Morino, Ryuji Otani, Muneyuki Kadota, Takayuki Ise, Hiroki Yamazaki, Kenya Kusunose, Koji Yamaguchi, Hirotsugu Yamada, Takeshi Soeki, Tetsuzo Wakatsuki, Daiju Fukuda, Mitsuharu Ueda, Masataka Sata

**Affiliations:** 1 Department of Cardiovascular Medicine, Tokushima University Graduate School of Biomedical Sciences, Tokushima, Japan; 2 Department of Community and Family Medicine, Tokushima University Graduate School of Biomedical Sciences, Tokushima, Japan; 3 Department of Neurology, Tokushima University Graduate School of Biomedical Sciences, Tokushima, Japan; 4 Department of Neurology, Graduate School of Medical Sciences, Kumamoto University, Kumamoto, Japan; 5 Department of Medical Genetics, Tokushima University Graduate School of Biomedical Sciences, Tokushima, Japan; 6 Department of Cardiology, Tokushima Red Cross Hospital, Tokushima, Japan; 7 Department of Cardiovascular Medicine, Osaka Metropolitan University Graduate School of Medicine, Osaka, Japan

**Keywords:** Genetics, Disease genetics

## Abstract

ApoA-I amyloidosis is an extremely rare form of systemic amyloidosis that commonly involves the heart, kidneys, and liver. ApoA-I amyloidosis is caused by amyloidogenic variants of *APOA1* that are inherited in an autosomal dominant manner. Here, we report a 69-year-old man with sporadic cardiac amyloidosis who was born to consanguineous parents and carried a homozygous variant of p.Leu202Arg in *APOA1*.

Amyloidosis is a group of diseases caused by the extracellular deposition of insoluble amyloid fibrils in various tissues and organs, including the heart, nerves, kidneys, and ligaments^1^. Hereditary amyloidosis is caused by genetic variants in amyloid precursor proteins, such as transthyretin (TTR) and apolipoprotein A-I (ApoA-I)^2^. ApoA-I amyloidosis is a rare autosomal dominant disorder, with more than 20 reported variants^3,4^. Here, we report a patient with cardiac ApoA-I amyloidosis who carried a homozygous variant of p.Leu202Arg in *APOA1*.

The patient was a 69-year-old man who was born to consanguineous parents and had no family history of heart disease. At 52 years of age, he developed dyspnea and edema in his extremities. A 12-lead electrocardiogram revealed a left bundle block and a first-degree atrioventricular block with low voltage. Echocardiography revealed a normal left ventricular (LV) diameter with a reduced ejection fraction of 46% and severe diffuse hypertrophy. Although diuretic therapy at age 53 temporarily relieved his symptoms, he was admitted to the hospital for the recurrence of heart failure (HF). On the basis of echocardiographic findings, cardiac amyloidosis was suspected; however, biopsy samples from the endocardium and gastrointestinal tract did not demonstrate amyloid deposition. At the age of 57 years, he underwent cavotricuspid isthmus ablation for an atrial flutter that triggered an HF relapse. At 58 years of age, he developed a complete atrioventricular block, and an implantable cardiac resynchronization therapy pacemaker was implanted. Over the subsequent years, the patient experienced multiple hospitalizations for recurrent HF. At 67 years of age, he presented to our hospital for further examination for HF.

A 12-lead electrocardiogram revealed a biventricular pacing rhythm. Chest radiography revealed cardiomegaly and lung congestion (Fig. 1a). Cardiac scintigraphy with technetium pyrophosphate (^99m^Tc-PYP) showed strong intensity in the myocardial region, with a heart-to-contralateral ratio of 2.0 (Fig. 1b). Echocardiography revealed a slightly enlarged LV diastolic diameter of 55 mm with a reduced ejection fraction of 40% and severe LV diffuse hypertrophy (interventricular septum thickness, 13 mm) accompanied by apical sparing of the longitudinal strain (Fig. 1c, d). Congo red staining of biopsy samples from the right ventricle revealed abundant amyloid deposits (Fig. 1e). Immunohistochemistry revealed no deposition of TTR or immunoglobulin light chain. Blood tests revealed slightly increased B-type natriuretic peptide (BNP, 154 pg/mL) and troponin I (31 pg/ml) levels. Monoclonal gammopathy was not detected. ApoA-I levels were normal (121 mg/dl), but HDL cholesterol (HDL-C) levels were decreased (35 mg/dL). Serum liver enzyme and creatine levels were within the normal range; albuminuria was not detected. A nerve conduction study revealed mild sensory polyneuropathy, which was compatible with amyloid neuropathy.

**Fig. 1:**
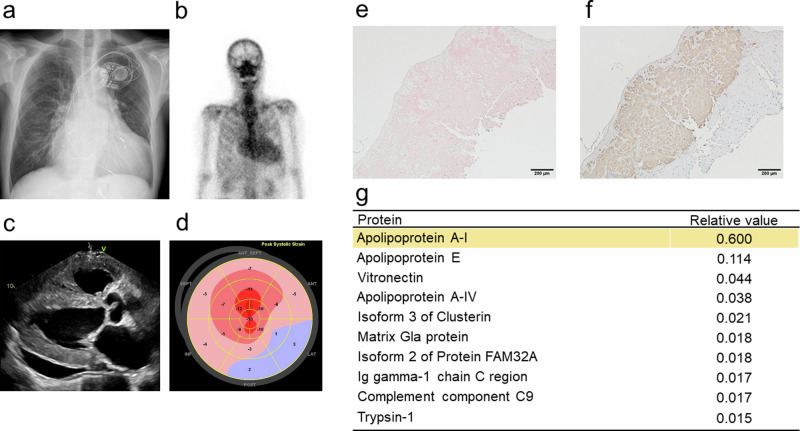
Clinical images and list of proteins detected in the heart. **a** Chest radiography image. **b** Cardiac scintigraphy with technetium pyrophosphate image. **c** Echocardiography image, long-axis view. **d** Echocardiography image, longitudinal strain. **e** Pathological images of Congo red staining of cardiac samples. **f** Image of immunohistochemistry with ApoA-I antibody. **g** List of proteins detected using laser microdissection‒liquid chromatography‒tandem mass spectrometry analysis of cardiac samples. The most abundant peptide was derived from ApoA-I.

We then performed laser microdissection‒liquid chromatography‒tandem mass spectrometry (LMD‒LC‒MS/MS) analysis to identify amyloidogenic proteins from formalin-fixed, paraffin-embedded cardiac tissue sections, as previously described^4,5^. The most abundant peptide was derived from ApoA-I (Fig. 1g); this result was confirmed by immunohistochemistry with an ApoA-I antibody (Fig. 1f).

Sanger sequencing of *APOA1* revealed that the patient had a homozygous variant of NM_000039.3:c.605 T > G, p.Leu202Arg (corresponding to p.Leu178Arg when excluding the signal peptide and propeptide) (Fig. 2a). p.Leu202 is located in the N-terminal region of ApoA-I and is highly conserved among species. The variant was classified as likely pathogenic (PP3 + PM1 + PM2 + PM5) according to the American College of Medical Genetics and Genomics (ACMG) classification. Multiple protein prediction algorithms indicated that this variant was damaging, with a CADD Phred score of 29.4. Moreover, several single-point variants were identified in N-terminal residues 194–202 of the protein^6^; previously, a different missense variant at this site, p.Leu202His, was determined to be pathogenic^7,8^. The patient’s older brother was heterozygous for the variant, whereas his older sister did not harbor it (Fig. 2b). Genetic testing was not performed for the other family members. The older brother showed no abnormal findings on electrocardiogram or echocardiography. Blood test results revealed normal BNP (25 pg/mL), slightly increased ApoA-I (158 mg/dl), and normal HDL-C (44 mg/dL) levels. Similarly, his older sister, who had a history of rheumatic arthritis, had normal electrocardiogram and echocardiography findings. Blood tests revealed normal levels of BNP (18 pg/mL) and increased ApoA-I (209 mg/dl) and HDL-C (93 mg/dL) levels.

**Fig. 2:**
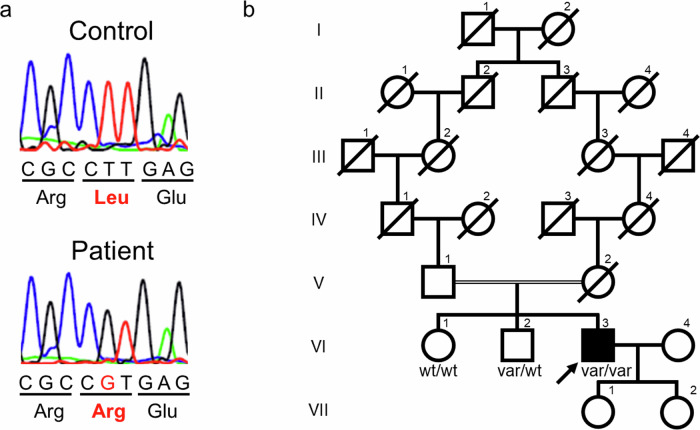
Sanger sequencing result and family pedigree. **a** Sanger sequencing analysis of *APOA1* revealed that the patient had a homozygous variant, p.Leu202Arg. **b** Family pedigree. wt wild type, var variant.

The patient continued diuretic treatment (40 mg of furosemide and 8 mg of torsemide) and achieved a stable disease course with a New York Heart Association functional classification II.

Hereditary amyloidosis comprises more than 30 subtypes, including TTR, ApoA-I, ApoA-II, gelsolin, lysozyme amyloidosis, cystatin C amyloidosis (ACys), fibrinogen Aα-chain, β2-microglobulin, ApoC2-II, and ApoC3^9^. Each subtype involves a different precursor protein and exhibits significantly varied clinical presentation. ApoA-I amyloidosis is an extremely rare form of systemic amyloidosis that commonly involves the kidneys, heart, and liver^1^. Genetically, ApoA-I amyloidosis is caused by amyloidogenic variants of *APOA1* inherited in an autosomal dominant fashion. After identification of the p.Gly50Arg variant in 1990^10^, more than 20 causative variants have been identified^11^. A basic study revealed that the ApoA-I N-terminal fragment is strongly associated with amyloid fibril formation, leading to systemic amyloidosis^12^. The majority of amyloidogenic mutations are located in two hotspot regions: the N-terminal region containing amino acid residues 50-131 and the C-terminal region containing amino acid residues 178-202 (corresponding to amino acids 26-107 and 154-178, respectively, when excluding the signal peptide and propeptide)^1^. Amyloid fibrils isolated ex vivo were found to be composed mainly of N-terminal fragments of ApoAI with lengths of 80–100 residues. N-terminal fragments of ApoAI enhance the formation of fibrils containing ApoAI fragments with lengths of 1–83 residues. On the other hand, the amyloid fibrils extracted from patients with C-terminal variants were composed mainly of fragments with lengths of 1–93 residues. Amyloidogenic mutations in the N-terminal region may increase protein flexibility in proximity to the putative cleavage site, releasing the N-terminal amyloidogenic domain^1^.

In addition, a biochemical study of the p.Leu202His variant, which occurs at the same amino acid position as the variant in this patient, demonstrated an increase in α-helical content but not β-strand content, accompanied by the formation of very short fibrils^11^. These biochemical properties are different from those observed in the classical variant p.Gly50Arg. The exact mechanisms by which the same phenotype develops from different genetic variants have not been fully elucidated. The amyloidogenic phenotype is related to altered structural features, including protein conformation and lipid binding, protein flexibility and stability, susceptibility to proteases, and aggregation propensity^1^. Variants in the N-terminal region frequently involve the kidney and liver, whereas those in the C-terminal region lead to cardiac, laryngeal, and cutaneous symptoms^6^. The difference in the structural properties of amyloid fibrils from different genotypic regions may also affect the phenotype.

For the patient in this study, the clinical picture and results of biochemical analysis were compatible with those of ApoA-I amyloidosis; however, the patient unexpectedly harbored a homozygous pathogenic variant, p.Leu202Arg, in the *APOA1* gene. Moreover, his older brother, who had a heterozygous p.Leu202Arg variant, did not exhibit amyloidosis-related symptoms at the age of 75 years. Generally, ApoA-I amyloidosis exhibits age-dependent penetrance. A survey of the p.Leu99Pro variant revealed that its penetrance was 38% at 60 years of age and 98.7% at 80 years of age or older^13^. Moreover, disease penetrance might be associated with the variant and the resulting amino acid substitution^6^. The incomplete and age-dependent penetrance of the *APOA1* variant could account for the presence of unaffected family members, especially the heterozygous older brother. Importantly, the proband inherited the disease in an apparent autosomal recessive manner, although ApoA-I amyloidosis is known to be an autosomal dominant disorder. Furthermore, there have been no reported cases of homozygous amyloidogenic *APOA1* variants. We speculate that the heterozygous p.Leu202Arg variant might have only a “weak” effect on amyloidogenesis and that the biallelic variant might be a prerequisite for symptom manifestation.

The p.Leu202Arg variant reported here causes an amino acid substitution in the C-terminal region. The region in which the amyloidogenic variant is located may be responsible for the genotypic differences. However, although we showed that the accumulant was derived from the ApoA1 protein, the biochemical characteristics of the accumulant and the pathophysiology of its accumulation remain unknown. Structural analysis of the protein produced by the mutant and studies using experimental animals are necessary to elucidate this pathophysiology in the future.

ApoA-I is the main apolipoprotein among plasma high-density lipoproteins (HDLs) with well-documented cardioprotective functions^3^. The reverse transport of cholesterol from peripheral tissues to the liver for excretion by promoting cholesterol efflux from tissues is a pivotal pathway and acts as a cofactor for lecithin cholesterol acyltransferase (LCAT). *APOA1* knockout mice presented normal HDL levels but altered HDL composition. Moreover, compared with control mice, knockout mice presented higher levels of triglycerides and free cholesterol and lower levels of cholesteryl ester (CE), suggesting that ApoA-I-deficient HDL is a poor substrate for hepatic lipase and LCAT, which may lead to decreased levels of HDL-C^14^. Patients with ApoA-I amyloidosis are reported to have decreased serum HDL-C and ApoA-I levels^15,16^. In our patient, the HDL-C level decreased, whereas his older brother with a heterozygous variant had normal HDL-C levels. This finding further suggests the additive effect of the biallelic p.Leu202Arg variant, which may reduce the function of ApoA-I and eventually decrease the level of HDL-C.

A noninvasive diagnostic criterion for cardiac TTR amyloidosis is applicable to most patients with amyloidosis^17^. If the results of bone scintigraphy with bisphosphonate are grade 2 or 3 with no monoclonal gammopathy, cardiac TTR amyloidosis can be diagnosed with > 99% sensitivity and 86% specificity. Although our patient met these criteria (grade 3 according to 99mTc-PYP scintigraphy and no monoclonal gammopathy), he was finally diagnosed with ApoA-I amyloidosis using LMD–LC‒MS/MS and genetic testing. The present case reaffirms the reduced specificity of nonbiopsy diagnostic criteria and the importance of biopsies for diagnosing TTR amyloidosis^2^. Moreover, this case also suggests that when immunohistochemistry results indicate a diagnosis other than TTR, LMD–LC‒MS/MS coupled with targeted gene sequencing can efficiently confirm an etiological diagnosis, even in the absence of a family history.

Currently, amyloid production or deposition in hereditary ApoA-I amyloidosis cannot be suppressed using any established treatment. Therefore, treatment exclusively focuses on relieving the symptoms of organ damage, such as the use of diuretics in patients with HF^1^. Although disease progression in ApoA-I amyloidosis is usually slow, its management often becomes very difficult once cardiac symptoms fully manifest. ApoA-I amyloidosis is extremely rare; therefore, it is not routinely included in the differential diagnosis of systemic amyloidosis^18^ and largely remains unexplored. Thus, the genotype‒phenotype correlation in ApoA-I amyloidosis needs to be further delineated to facilitate early diagnosis, effective genetic counseling, and risk stratification of pathogenic variant carriers.

## HGV Database

The relevant data from this Data Report are hosted at the Human Genome Variation Database at 10.6084/m9.figshare.hgv.3416.
